# Genome Sequence of *Pseudomonas plecoglossicida* Strain NZBD9

**DOI:** 10.1128/genomeA.01412-17

**Published:** 2018-01-25

**Authors:** Lixing Huang, Lingmin Zhao, Yongquan Su, Qingpi Yan

**Affiliations:** a Fisheries College, Key Laboratory of Healthy Mariculture for the East China Sea, Ministry of Agriculture, Jimei University, Xiamen, Fujian, People’s Republic of China; b State Key Laboratory of Large Yellow Croaker Breeding, Ningde, Fujian, People’s Republic of China

## Abstract

*Pseudomonas plecoglossicida* NZBD9 is the causative agent of white nodules in cultured large yellow croaker in Fujian Province, China. We sequenced the genome of NZBD9 to gain a better understanding of the etiological agent. The genome sequence of the bacterium consists of 5.44 million bp, with a G+C content of 61.9%.

## GENOME ANNOUNCEMENT

*Pseudomonas plecoglossicida* is the etiological agent of bacterial hemorrhagic ascites (BHA) in freshwater fish and was first isolated from ayu (*Plecoglossus altivelis*) in Wakayama Prefecture, Japan, in 1991 (1). To date, infection of ayu, pejerrey (*Odontesthes bonariensis*), rainbow trout (*Oncorhynchus mykiss*), and large yellow croaker (*Pseudosciaena crocea*) with *P. plecoglossicida* has been reported (1–3). Outbreaks of *P. plecoglossicida* infectious disease in cage-farmed large yellow croaker, characterized by white nodules in the spleen, kidney, and liver of a diseased fish and results in high mortality, have had a severe economic impact on fisheries in the Fujian and Zhejiang provinces in China (1).

In our previous study, a strain of *P. plecoglossicida* (NZBD9) was isolated in Fujian Province in April 2013 from internal organs of diseased *P. crocea* with typical symptoms of white nodules (4). Artificial infection of *P. crocea* with NZBD9 could cause the symptoms in the internal organs and result in high mortality. In the current study, in order to gain a better understanding of this etiological agent, we sequenced the *P. plecoglossicida* NZBD9 genome using the Ion Proton system.

Genomic DNA from NZBD9 was extracted using a QIAsymphony SP instrument with the QIAsymphony DSP DNA kit (Qiagen, Hilden, Germany). The NZBD9 genome was sequenced, assembled, and finished at CapitalBio Technology (Beijing, China) using the Ion Proton system with the Ion PI sequencing 200 kit version 2 chemistry (200-bp read length; Life Technologies, Inc.). A total of 3,888,623 reads were generated. Genome sequences were assembled as 331 scaffolds, with an *N*_50_ length of 61,164 bp, a total length of 5,435,598 bp, and a G+C content of 61.9%. The scaffolds range in length from 60 bp to 197,236 bp. The genome sequence was annotated using the databases Rapid Annotations using Subsystems Technology (RAST) (5) and Prokaryotic Genome Annotation Pipeline (PGAP) (https://www.ncbi.nlm.nih.gov/genome/annotation_prok/), which revealed many virulence factors, including the iron uptake system, type III and type VI secretion systems, type IV pili, and multiple flagellins (6–8).

*P. plecoglossicida* NB2011, the causative agent of white nodules in cultured *P. crocea* in Zhejiang Province, China, was sequenced in 2013 (2) and comprises 5.41 million bp, with a G+C content of 62.8%. In order to compare these two strains, reads from NZBD9 were aligned against the genome sequence of NB2011 using the Blast-plus-2.2.30 software. Alignment preprocessing steps and variant calling were done according to the Genome Analysis Toolkit (GATK) best practices guidelines (9). This analysis showed that the similarity between the NZBD9 and NB2011 genomes was up to 99.76%. This indicated that the NZBD9 genome sequence is very similar to the NB2011 genome sequence and that they can be considered the same species at the taxonomic level. Genome analysis of NZBD9 also revealed structural differences, such as the presence of 56 and 107 structural variations and single-nucleotide polymorphisms (SNPs), respectively, compared with the sequence of NB2011 (2).

The genome information of NZBD9 presented in this study will be useful for further studies, such as the analysis of its role as etiological agent, with the aim of investigating the molecular mechanisms of white nodules.

### Accession number(s).

This whole-genome shotgun project has been deposited at DDBJ/ENA/GenBank under the accession number PHNR00000000. The version described in this paper is version PHNR01000000.
